# Advancing critical care: time to kiss the right frog

**DOI:** 10.1186/cc11501

**Published:** 2013-03-12

**Authors:** Mervyn Singer

**Affiliations:** 1 Department of Intensive Care Medicine, Bloomsbury Institute of Intensive Care Medicine, University College London, Cruciform Building, Gower Street, London WC1E 6BT, UK

## Abstract

The greatest advances in critical care over the past two decades have been achieved through doing less to the patient. We have learnt through salutary experience that our burgeoning Master-of-the-Universe capabilities and the oh-so-obvious stratagems instilled in us from youth were often ineffective or even deleterious. This re-education process, however, is far from complete. We are now rightly agonizing over the need for better characterization of pathophysiology, earlier identification of disease processes and a more directed approach to therapeutic intervention. We need to delineate the point at which intrinsic and protective adaptation ends and true harmful pathology begins, and how our iatrogenic meddling either helps or hinders. We need to improve trial design in the heterogeneous populations we treat, and to move away from syndromic fixations that, while offering convenience, have generally proved counterproductive. Importantly, we need to discover a far more holistic approach to patient care, evolving from the prevailing overmedicalized, number-crunching perspective towards a true multidisciplinary effort that embraces psychological as well as physiological well-being, with appropriate pharmacological minimization or supplementation. Complacency, with an unfair apportion of blame on the patient for not getting better, is the biggest threat to continued improvement.

## 

Critical care medicine has reached an important crossroads. The discipline's infancy and adolescence revolved around a 'can do, will do' attitude. Physiology and biochemistry could be tweaked, manipulated or bullied into submission. We deferentially followed the seemingly unassailable logic that normal healthy values would provide the optimal milieu for either maintaining organ function or hastening recovery. Short-term and long-term complications of critical illness, including death, were usually related to the acute disease process and/or the patient's underlying poor protoplasm. It was easy and convenient to accuse patients for their subsequent demise or less-than-perfect survival; we could blame the severity of their sepsis or acute respiratory failure, their underlying cancer or diabetes, or their lifestyle abuses from excess alcohol, smoking, recreational drugs and obesity. We were heroic and saintly figures, fighting unseen mediators and recalcitrant surgeons, railing against incompetence on general wards and administrative dereliction in failing to provide the latest fancy life-saving apparatus or expensive drug. We occasionally generated problems such as misplaced catheters or untoward drug interactions, but multiple pneumothoraces, pressure sores and nosocomial infections were viewed as acceptable and perhaps even unavoidable prices to pay for our last-ditch fight against the Grim Reaper on behalf of these desperately ill people.

We also embraced the multicenter trial as the best form of evidence and placed it on a lofty pedestal for worship. To satiate this particular idol by achieving the necessary sample size of sacrificial virgins, we lumped patients into artificial syndromes (severe sepsis, acute respiratory distress syndrome, acute kidney injury) - usually irrespective of the underlying etiology and other important disparities. We included patients who were often too well or too sick to benefit, yet they fulfilled trial entry criteria and we could therefore recruit the required numbers. We were reassured that randomization would iron out discrepancies in populations and concurrent therapies, but paid little notice to other factors such as disease trajectory that may crucially impact upon outcome.

With the increasing maturity of the specialty, we began to recognize our foibles and our misplaced enthusiasms. Our life-saving stratagems more often than not contributed to the patient's demise, or to their short-term and long-term morbidity. We discovered that we overventilated, overfluidized, overfed, overtransfused and over sedated, and that these all contributed significantly to harm [1-6]. Although I have just disparaged large-scale randomized controlled trials, they have nevertheless been the main drivers of change, particularly as the negative findings relating to current practice often went against the seemingly logical rationale that informed the study design. Yet does this mean that high-dose corticosteroids are not always bad [7], that transfusion to a high hemoglobin target may be appropriate in certain cases, that activated protein C is life-saving in the right patient, and that heavy sedation may sometimes be indicated? We have to reconcile evidence-based medicine that is applicable to populations, with the optimal treatment for an individual patient at a particular point of time in their acute illness.

We are slowly extending our appreciation of widespread iatrogenic harm to other procedures routinely performed within the ICU. For example, the detriment associated with excess use of inotropes and antibiotics is being increasingly recognized [8], although not necessarily acted upon. Our youthful dalliances and indiscretions are being replaced with a more sober perspective and a more measured and mature approach, yet we still remain prone to lapses and continued denials. For example, gastric acid suppressants [9,10] and infection control procedures [11,12] are likely to adversely affect patient outcomes, yet their routine use is barely challenged at present. The rollercoaster of corticosteroid use will probably continue until we can more precisely define who and when to treat, with how much and for how long.

We have kissed lots of frogs, but are still waiting to find the true prince. Identifying the right frog may require better diagnostics and biomarkers than we have at present. Or, perhaps, it is not frogs we should be kissing in the first place. The necessary changes in direction may be conceptual or technological. Our current management paradigms may ultimately prove to be misguided, or lacking the necessary tools to select and tailor the right amount of a specific treatment to the individual, and avoid/minimize those that harm. This sophistication is imperative to rekindle the interest of drug companies who have been dissuaded from investment in new sepsis research by a litany of repeated trial failures.

So where do we go from here? Outside therapeutic hypothermia [13,14], I struggle to think of a recent specific intervention that has categorically made a large difference to outcomes. However, even the effectiveness of this procedure has been challenged in patients suffering cardiac arrest not related to ventricular fibillation [15,16]. On the contrary, a mortality/morbidity impacthas been achieved by stopping/moderating previous excess, whether it be large tidal volumes [1], too much blood or fluid [2,4], undue sedation [5,6] or overfeeding [3].

Perhaps we should reconfigure our understanding of disease pathology in the light of adaptation (or failed adaptation) of body systems. Therapeutic hypothermia involves decreasing cerebral metabolism as a protective strategy. Therapeutic hypothermia has also been applied, albeit with less consistent success, to head injury [17] and myocardial revascularization [18]. Organ dysfunction may actually represent a reconfiguration of cellular priorities away from processes normally undertaken in health towards dealing with prolonged and severe inflammation and/or ischemia. The concept of myocardial hibernation is well enshrined within cardiology. Here, ongoing myocardial hypoperfusion - sufficient to impair normal functioning yet not induce immediate infarction - triggers a decrease in contractility. This reduces metabolic demands, thereby protecting the vulnerable muscle. Importantly, this hypofunctionality is reversible upon restoration of adequate perfusion.

So why does the same not hold true for other organs involved in multiorgan failure where minimal cell death is apparent? This could apply, for example, to acute kidney injury where the major energetic requirement in health is reabsorption of 98% of glomerular filtrate. Impaired renal perfusion leading to tissue hypoxia would thus result in a massive and undesirable polyuric fluid loss, and so the kidney sensibly switches itself off to minimize glomerular filtration, and switches on again once the insult has passed [19]. What about liver dysfunction? Here, the decrease in metabolizing capacity and biliary transport increases plasma bilirubin that, in turn, offers significant antioxidant capacity [20]. Perhaps, in severe inflammatory states, this substitutes for the falling plasma levels of another potent circulating antioxidant, albumin, due to decreased hepatic production and increased transcapillary movement. A high blood lactate, long perceived to be deleterious due to its association with illness severity and death, is now also recognized to be potentially adaptive, offering vital organs such as the brain, heart and liver an alternative and important substrate for energy production [21,22]. Hypercapnia and hypoxemia also trigger an array of protective responses [23,24] that may be abrogated through aggressive correction.

These proposed intrinsic adaptations need to be placed in the context of an untreated critically ill patient who, in the era preceding modern medicine, would not have received liters of fluid resuscitation, nor heavy sedation to blunt potentially protective reflexes and neural regulatory networks, nor a battery of drugs that require metabolism and excretion. Multiorgan failure may therefore provide late-stage protection and the potential to recover, provided the adaptations do not become maladaptive [25]. Yet our continued use of 'failure' terminology generates a perhaps undeservedly negative connotation and a flawed fixation on correcting seemingly peculiar numbers that the body arguably does not want fixed. The corollary of inadvisable overcorrection of physio-biochemical abnormalities plus toxicity from high levels of unwanted drugs and nutrients that cannot be metabolized/excreted is a further increase in body stress that synergizes with the host of other stressors inflicted on the sick patient [26].

If we attach some credence to this alternative paradigm of critical illness, then this should also trigger a reevaluation of how we manage our patients. Clearly, this should include avoidance or, at least, minimization of unneeded or excessive drugs and interventions, plus targeted mobilization regimens that attend to both the psychological and physiological needs of the patient. It is axiomatic that the acute phase of critical illness has essentially resolved within a few days yet we not infrequently have to wait weeks or even months for survivors to recover enough independent organ function to cerebrate, breathe, move and urinate adequately.

Is this delayed resolution simply a function of their acute illness, or do our current therapies add significantly to this problem [26]? Recovering patients may fester in bed for too long, accelerating bone and muscle loss. Vascular access may be unnecessarily kept *in situ*, enhancing the risk of secondary infection. Do frequent changes of patient position (assisted by inappropriate ventilator patterns) propagate bilateral spread of infected lung secretions beyond the natural local defense strategy of lobar collapse and consolidation [27]? Does hemofiltration delay natural renal recovery, inducing a self-perpetuating dependency? We inflict major stress - be it pharmacological (for example, with catecholamines), physiological (for example, by excessive rehabilitation or over-rapid weaning), and psychological - through unnecessary pain and discomfort, sleep deprivation, anxiety, boredom, and communication failure. Prolonged stress - physiological, pharmacological and/or psychological - contributes to myocardial depression (for example, Takotsubo cardiomyopathy), immune suppression with stimulation of bacterial growth and virulence, metabolic inefficiency, glucose intolerance, hypercortisolism, muscle catabolism, agitation and delirium, and a marked prothrombotic tendency [8,26].

Awareness of the negative whole-body impact of excessive stress stretches back approximately 80 years to the pivotal work by Hans Selye [28]. He inflicted a variety of insults on rats, including drugs, spinal cord transection, temperature changes and excess exercise and demonstrated identical pathological changes. If this concept is valid then, logically, we should be striving energetically to alleviate stress. The therapeutic approach will involve directed pharmacology - for example, β-blockade [29], α_2_-agonism [30], or judicious night-time alcohol selected by patient preference - plus attention to the patient's environment and psychological well-being [31,32], appropriate use of nutritional volumes and contents, and targeted mobilization regimens to avoid stressful fatigue yet make appropriate progress in terms of strengthening both mind and body.

What about novel therapies? Which strategic direction should be adopted? To my mind there are three obvious unmet needs.

Firstly, many of the drugs that have been trialed and discarded undoubtedly have merit if given for the right indication to the right patient, at the right time, in the right dose, and for the right duration. The heterogeneity of sepsis itself, and the broad population it variably affects, mandate a more tailored approach. Theranostics and personalized medicine will come to critical care in similar fashion to the far better-resourced oncology arena. The link between human epidermal growth factor receptor-2 positivity of breast cancer and a beneficial response to herceptin [33] is the best-known example of a burgeoning field that includes imaging biomarkers (for example, for use in magnetic resonance imaging and nuclear medicine), diagnostic/prognostic protein biomarkers (for example, involving immunohistochemistry, immunoassays and labeled antibiotics), molecular diagnostics (such as PCR, quantitative PCR, DNA sequencing and microarrays), cell-based biomarkers (identifiable by fluorescence-activated cell sorting) and drug efficacy response biomarkers (based, for example, on genes, proteins, metabolites).

Secondly, preventive medicine needs to come more to the fore. Although far less glamorous, it is indisputably worthwhile provided it is performed in a cost-effective manner. In the critical care unit in which I ply my trade, both junior and senior doctors alike groan at the large throughput of relatively well high-risk surgical patients spending their first postoperative days with us for optimization of fluids, breathing, pain relief and mobilization, and early identification and treatment of any early complication. This prevention/early intervention ethos is extended to intraoperative circulatory optimization, and to outreach teams actively scouring the surgical and medical wards for patients with early signs/symptoms of deterioration, as well as regular follow-up of those discharged from critical care. The relative ordinariness of such practice compared with the high-octane environment surrounding a critically ill patient does, however, translate into excellent results that place our hospital at the forefront of published UK outcomes with mortality rates 25 to 30% below the national average [34]. Preventive medicine will also be extended by improved diagnostics for infection and early sepsis. These will be point-of-care devices offering results within 1 to 2 hours rather than 1 to 2 days, and such tools are already becoming commercially available [35]. This will enable targeted interventions that both prevent patients from spiraling into organ dysfunction and also avoid the use of unnecessary and injurious antibiotics.

Thirdly, we need to look beyond direct modulation of systemic inflammation and towards other targets. The repeated multicentre trial failures of agents targeted against suppressing the inflammatory response [36], starting with anti-endotoxin strategies in the 1990s through to the withdrawal of activated protein C in 2011, forcefully indicate the need for better selection of suitable patients, optimal timing and titrated dosing using drugs or techniques offering similar biologic rationales. On the contrary, it may be beneficial to stimulate the immune response in appropriate patients with evidence of immunosuppression, as has been shown in limited trials to date with granulocyte-macrophage colony-stimulating factor [37,38]. Attention should perhaps be directed away from the inflammatory response and the immune system towards endocrine, metabolic and bioenergetic targets [39,40]. Excessive damage or inhibition could be attenuated or even prevented through agents with pleiotropic properties such as estrogen and statins, or with directed mitochondrial antioxidants to prevent dysfunction of the predominant energy-producing apparatus of most cell types. Conversely, recovery from organ failure could be enhanced by stimulating regeneration of healthy, functioning mitochondria with stimulators of biogenesis, or of muscle mass and strength with a combination of exercise programs and anabolic steroids.

Since its infancy, critical care has come a long way. From being let loose in the toyshop and throwing occasional tantrums, through the teenage years of gawky charm yet sporadic bursts of overconfidence or petulance, it is gradually blossoming into taking a more well-rounded and considered approach yet still retaining the fundamental energy and drive belonging to a new specialty. This freshness also needs to be utilized to continue to challenge dogma and revisit accepted paradigms that are often based on rather shallow and rocky foundations.

## Abbreviations

PCR: polymerase chain reaction.

## Competing interests

The author declares that they have no competing interests.

## Declarations

This article has been published as part of *Critical Care *Volume 17 Suppl 1, 2013: Future of Critical Care Medicine. The supplement was proposed by Fresenius Kabi based on presentations from the 'Future of critical care medicine (FCCM) 2012: Today's practice and a look to the future' symposium. Articles were commissioned by the journal, were independently prepared by the authors and have been peer reviewed by the journal. The supplement was edited by John Marini, who declares that he has no competing interests. Publication of the supplement was supported by Fresenius Kabi.
